# Does topical application of tranexamic acid reduce intraoperative bleeding in sinus surgery during general anesthesia?^☆^

**DOI:** 10.1016/j.bjorl.2019.08.006

**Published:** 2019-10-03

**Authors:** Haram Kang, Se Hwan Hwang

**Affiliations:** The Catholic University of Korea, College of Medicine, Department of Otolaryngology-Head and Neck Surgery, Seoul, Republic of Korea

**Keywords:** Tranexamic acid, Endoscopic sinus surgery, Operative bleeding, Systematic review, Meta-analysis, Ácido tranexâmico, Cirurgia endoscópica do seio nasal, Sangramento operatório, Revisão sistemática, Metanálise

## Abstract

**Introduction:**

Tranexamic acid is a hemostatic agent, which inhibits fibrin degradation, which may be beneficial in controlling bleeding during surgery.

**Objectives:**

The purpose of this study was to provide a meta-analysis and review of the effects of tranexamic acid on hemorrhage and surgical fields and side effects on patients during endoscopic sinus surgery.

**Methods:**

Two authors independently searched six databases (Medline, Scopus, Embase, Web of Science, Google Scholar and Cochrane library) from the start of article collection until July 2018. Postoperative complications such as intraoperative bleeding, operative time, hypotension, nausea, vomiting, and coagulation profile were included in the analysis of tranexamic acid (Treatment Group) and placebo (Control Group) during the operation.

**Results:**

The amount of blood loss during surgery was statistically lower in the treatment group compared to the placebo group, and the surgical field quality was statistically higher in the treatment group than in the placebo group. On the other hand, there was no significant difference in operation time, hemodynamics, or coagulation profile between groups. In addition, tranexamic acid had no significant effect on vomiting and thrombosis compared to the Control Group.

**Conclusion:**

This meta-analysis has shown that topical administration of tranexamic acid can reduce the amount of bleeding during surgery and improve the overall quality of the surgery. Hemodynamic instability during surgery, vomiting after surgery, or abnormal clotting profile were not reported. Additional studies are needed to confirm the results of this study because there are fewer studies.

## Resumo

**Introdução:**

O ácido tranexâmico é um agente hemostático, que inibe a degradação da fibrina e pode ser benéfico no controle do sangramento durante a cirurgia.

**Objetivos:**

Fazer uma metanálise e revisão dos efeitos do ácido tranexâmico na hemorragia e nos campos cirúrgicos e efeitos colaterais em pacientes durante a cirurgia endoscópica do seio nasal.

**Método:**

Dois autores realizaram independentemente uma busca em seis bancos de dados (Medline, SCOPUS, Embase, Web of Science, Google Scholar e Cochrane) desde o início da coleta de artigos até julho de 2018. Complicações pós-operatórias como sangramento intraoperatório, tempo operatório, hipotensão, náusea, vômitos e perfil de coagulação foram incluídos na análise do ácido tranexâmico (grupo de tratamento) e placebo (grupo controle) durante a cirurgia.

**Resultados:**

A quantidade de perda de sangue durante a cirurgia foi estatisticamente menor no grupo de tratamento comparado com o grupo placebo e a qualidade do campo cirúrgico foi estatisticamente maior no grupo de tratamento do que no grupo placebo. Por outro lado, não houve diferença significante no tempo cirúrgico, hemodinâmica ou perfil de coagulação entre os grupos. Além disso, o ácido tranexâmico não teve efeito significante na ocorrência de vômitos e trombose em comparação ao grupo controle.

**Conclusão:**

Esta metanálise mostrou que a administração tópica de ácido tranexâmico pode reduzir a quantidade de sangramento durante a cirurgia e melhorar a qualidade geral dela. Instabilidade hemodinâmica durante a cirurgia, vômitos após a cirurgia ou perfil de coagulação anormal não foram relatados. Estudos adicionais são necessários para confirmar os resultados desta pesquisa, porque há poucos estudos na literatura.

## Introduction

Nasal bleeding during endoscopic sinus surgery may increase the danger of airway obstruction through the aspiration of blood clots,^1^ which would support the use of general anesthesia during nasal surgeries.^2^ However, general anesthesia can reduce small vessel resistance and worsen hemorrhagic tendency during surgery.^3^ Because of the narrow space in the nasal cavity, bleeding during surgery can cause a significant decrease in visual acuity during surgery, which increases the risk of complications during surgery, increases the operation time and can make the operation incomplete. Various methods have been used to attempt to reduce intraoperative bleeding loss to ensure better visualization of the surgical field, including controlled hypotension, use of topical vasoconstrictors, preoperative steroid administration and others.

Tranexamic acid functions as the competitive antagonist at the lysine site on plasminogen.^4^, ^5^ During all surgical procedures, the tissue plasminogen activator is released due to tissue damage during surgery, which can convert tissue plasminogen to plasmin, promote fibrinolysis and activate the fibrinolytic system. Thus, tranexamic acid functions as an anti-fibrinolytic agent by inhibiting the tissue plasminogen activator.^6^ It can be applied topically or systemically with this mechanism in the coagulation cascade to reduce intraoperative bleeding.^4^ The results of several recent studies on endoscopic sinus surgery through topical administration of tranexamic acid are encouraging in terms of the efficacy of tranexamic acid for intraoperative bleeding and other pathological conditions.^6^, ^7^, ^8^ The purpose of this study is to analyze the efficacy of tranexamic acid to improve the surgeon and patient experiences of sinus surgery.

## Methods

### Search strategy and selection of studies

Researches published in English before February 2018 using terms such as “endoscopic sinus surgery”, “tranexamic acid”, “general anesthesia”, “Surgical area”, “Nausea and vomiting”, “Thromboembolism” and “Hypotension” were published in Medline, SCOPUS, Web of Science, Embase, Cochrane library, and Google Scholar.

**Figure 1:**
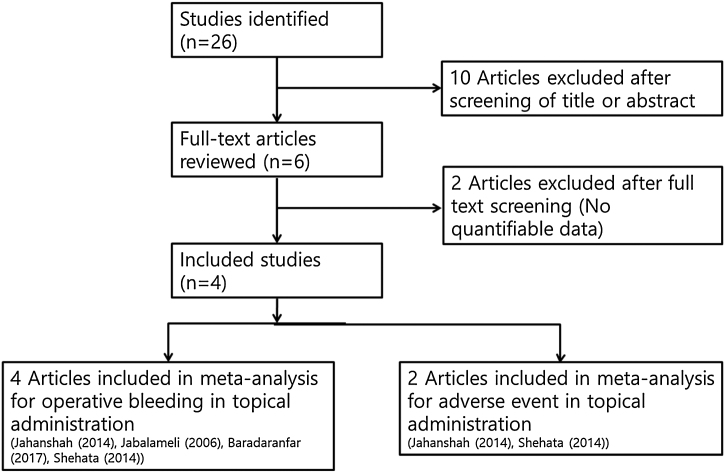
Diagram of selection of studies.

Two independent reviewers checked all abstracts and titles for candidate studies and excluded studies that were not associated with the pre-operative topical application of tranexamic acid. If the decision on inclusion could not be made by the abstract alone, we could get the full text of the research potentially relevant to the topic. A randomized controlled trial that meets the following inclusion criteria is subject to review: trials studying patients undergoing endoscopic sinus surgery and local administration of tranexamic acid perioperative administration. Studies were excluded if, in addition to sinus surgery, the patient had undergone other operations such as turbinoplasty, adenoidectomy, or if multiple reports were based on the same test data. If there was missing or incomplete data, an attempt had been made to obtain more detailed information directly from the author. If the results we were interested in were not obviously reported as quantifiable data, or if the proper data could not be identified, the study was excluded from the analysis (Fig. 1).

### Data extraction and risk of bias assessment

The measured outcomes were intra-operative blood loss,^4^, ^6^, ^7^, ^9^ surgical field score,^4^, ^9^, ^10^, ^11^ operative time,^4^, ^7^, ^9^ intra-operative blood pressure,^4^, ^7^ postoperative coagulation profiles,^4^, ^7^ the occurrence (incidence or percentage of patients) of post-operative nausea and vomiting^4^, ^7^ and thrombotic accident.^4^, ^7^ These results were compared between the tranexamic acid group treated and the control group treated with default saline or additional hemostatic topical agents (the same condition with intervention group without the application of tranexamic acid). The number of patients, grade used, intra-operative and operative results, incidence or percentage of adverse events, and measured p-values were abstracted to compare the effect of tranexamic acid with control groups. The risk of bias for each trial was assessed utilizing the Cochrane Risks of bias tool.

### Statistical analysis

The “R” statistical software (R for Statistical Computing, Vienna, Austria) was used to conduct meta-analysis of the enrolled studies. If the raw data were presented as a continuous variable, a meta-analysis was conducted using the Standardized Mean Difference (SMD). This method was chosen to measure the effect size since there is no standardized measure in all studies (intraoperative blood loss, operative field score, operative time, intraoperative blood pressure and postoperative clotting profile). The outcome of the incidence analysis was conducted using the Odds Ratio (OR). Sensitivity analysis was performed to assess the impact of each study on the outcome of the overall meta-analysis.

## Results

Four studies of 226 participants were included in this study. The risks of bias and study characteristics were presented in Table 1. We did not evaluate the publication bias because the number of trials included was not adequate to conduct funnel plots or advanced regression analysis.

**Table 1:** Summary of studies included in the meta-analysis.

Study (year)	Sample size	Study design	Comparison	Outcome measure analyzed	Risk of Bias of randomized studies
Jahanshahi (2014)	60	Randomized controlled study	Three pledgets soaked with tranexamic acid 5% and phenylephrine 0.5% for 10 minutes in each nasal cavity before surgery vs. three pledgets soaked with only phenylephrine 0.5%	Operative bleeding	Risk of Bias (low risk)
				Surgical field quality	
				Intraoperative blood pressure	
				Coagulation profile (PT and PTT)	
				Adverse effect (postoperative nausea and vomiting, thromboembolism)	
Jabalameli (2006)	56	Randomized controlled study	Tranexamic acid (1000 mg diluted in 20 mL normal saline) administered topically vs. saline	Operative bleeding	Risk of Bias (low risk)
				Surgical field quality	
Baradaranfar (2017)	60	Randomized controlled study	Tranexamic acid 2 g mixed in normal saline with a total volume of 400 mL for irrigation vs. saline	Operative bleedin	Risk of Bias (low risk)
				Surgical field quality	
Shehata (2014)	50	Randomized controlled study	Topical tranexamic acid (1000 mg diluted in 20 mL normal saline) for packing and irrigation vs. warm saline vs. saline	Operative bleeding	Risk of Bias (unclear risk)
				Surgical field quality	
				Intraoperative blood pressure	
				Coagulation profile (PT and PTT)	
				Adverse effect (postoperative nausea and vomiting, thromboembolism)	

### Tranexamic acid compared to control for intraoperative outcomes

**Figure 2:**
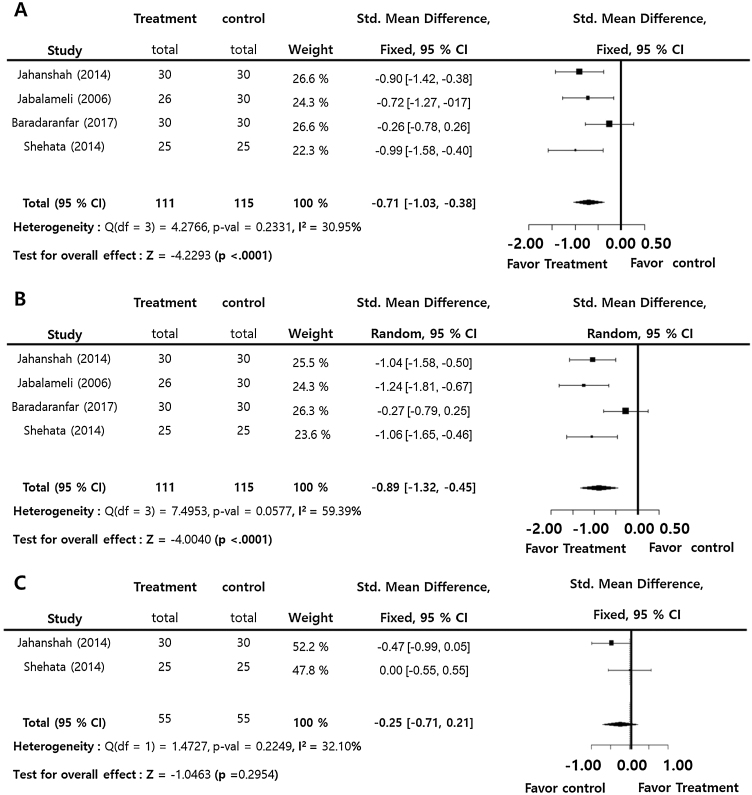
Perioperative tranexamic acid vs. control. Standard mean difference of intra-operative blood loss (A), surgical field score (B), operative time (C) and intra-operative blood pressure.

Intra-operative blood loss (SMD = −0.71; 95% Confidence Interval −95% CI: −1.03 to −0.38, I^2^ = 30.95%) and surgical field score (SMD = −0.89; 95% CI: −1.32 to −0.45, I^2^ = 59.39%) were statistically significantly lower in the treatment group compared to the control group. On the contrary, there was insignificant difference in intra-operative blood pressure (SMD = −0.29; 95% CI: −1.51‒0.94, I^2^ = 93.22%) between the groups. Intra-operative blood pressure also showed no significant difference (SMD = −0.25; 95% CI: −0.71‒0.21, I^2^ = 32.10%) between the groups. There were insignificant inter-study heterogeneities (I^2^ < 50) in the outcomes except for operative time and surgical field score (Fig. 2).

### Tranexamic acid compared to control for postoperative side effects such as emesis and coagulation profiles

**Figure 3:**
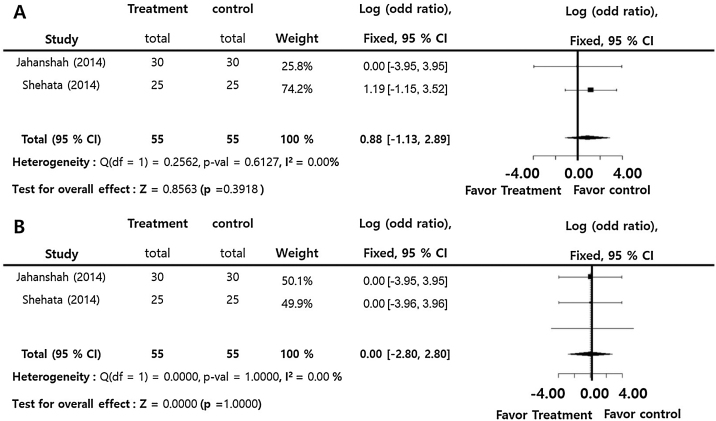
Perioperative tranexamic acid vs. control. Odds ratios of the incidence of postoperative nausea and vomiting (A) and thrombotic accident (B).

The incidences of postoperative nausea and vomiting (log OR = 0.88; 95% CI: −1.15‒2.89, I^2^ = 0.00%) and thrombotic accident (log OR = 0.00; 95% CI: −2.80‒2.80, I^2^ = 0.00%) did no significantly differ between two groups. There was insignificant inter-study heterogeneity (I^2^ < 50) in the overall outcomes (Fig. 3).

**Figure 4:**
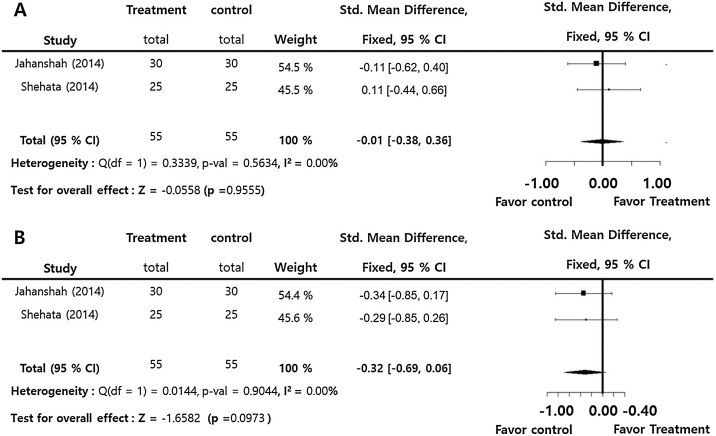
Perioperative tranexamic acid vs. control. Standard mean difference of prothrombin time (A) and partial thromboplastin time (B).

Prothrombin time (SMD = −0.01; 95% CI: −0.38‒0.36, I^2^ = 0.00%), and partial thromboplastin time (SMD = −0.32; 95% CI: −0.69‒0.06, I^2^ = 0.00%) presented insignficant differences between the groups. There were insignificant inter-study heterogeneities (I^2^ < 50) in the overall outcomes (Fig. 4).

### Sensitivity analyses

Sensitivity analysis repeated the meta-analysis, omitting different studies each time, to assess the differences in the combined estimates. All results match the above results.

## Discussion

Local tissue injury and administration of crystalloid like fluid during surgery could lead to the activation of fibrinolysis, which is known to be related to intra-operative blood loss and poor surgical field visibility.^10^ Tranexamic acid is a synthetic derivative of amino acid lysine that blocks fibrinolysis by blocking the lysine binding site of plasminogen and inactivating plasminogen and inhibiting fibrin binding to plasminogen.^11^ Until now, there have been several trials into the effect of tranexamic acid on nasal surgery.^5^, ^12^, ^13^, ^14^ Previous study conducted to collect data from these studies, and they found that tranexamic acid was beneficial in reducing intra-operative blood loss and improving the quality of the surgical field for endoscopic sinus surgery.^15^

Nevertheless, there were two important methodologic problems in the previous review by Pundir et al.,^15^ which enrolled five studies to increase the number of trials to the maximum, but I found that the authors collected data regardless of how they were applied. First, three out of five included studies investigated the effect of systemic application, while two studies analyzed the efficacy of topical application. The outcomes were analyzed and presented by summing the results from all enrolled studies regardless of administration type. Second, most studies included study group patients and control group patients separately. In contrast, ten patients were enrolled and two nostrils of a single patient were divided into two groups of nostrils of one study nostril and control nostril and the data were analyzed as the study enrolled 20 patients in the previous meta-analysis.^8^ Due to the heterogeneity of administration types and patient enrollment, meta-analysis could be difficult and results would become unreliable. Additional research has been published since the publication of the previous meta-analysis.^5^, ^11^, ^12^, ^13^, ^16^, ^17^ The aforementioned reasons necessitate our current meta-analysis.

Tranexamic acid has been administered topically or intravenously in various surgeries or clinical situations such as bleeding diatheses. However, the systemic use of it has mainly gastrointestinal adverse effects, including postoperative nausea and vomiting with the incidences of these ranging from 10% to 20%.^8^ A rapid intravenous bolus administration can develop significant hypotension.^18^ Furthermore, although tranexamic acid is reported to be tolerable and safe, the risk of thromboembolism has traditionally raised concerns about its use.^19^, ^20^ On the contrary, topical use of tranexamic acid can benefit locally from a lower dose requirement and a predicted reduction in systemic absorption that may reduce the risk of systemic side effects. Clinically, topical use has already been used in various kinds of surgical procedures including sinus surgery. Therefore, in this study, we included studies with perioperative topical administration methods for sinus surgeries under general anesthesia.

Our results showed that intraoperative bleeding and visual field visibility improved statistically in treatment (tranexamic acid) group compared to placebo group. Bleeding control during surgery is known to be one of the most important factors in improving successful surgical opportunities. Because the scope of surgery in the nasal cavity is narrow, mild hemorrhage can distort the view of the endoscope and cause significant structural damage, prolongation of operation or even incomplete surgery.^7^ This may result from the local antifibrinolytic effect of the drug as opposed to the local fibrinolysis caused by a natural event leading to bleeding.^6^ Previous meta-analyses showed that topical or systemic tranexamic acid reduces bleeding during surgery and improves the quality of the surgical field,^15^ which was consistent with our results. In contrast, the operative time did not decrease significantly in the treatment group. Theoretically, intra-operative bleeding causes multiple interruptions during the surgery for suctioning and packing and increases surgical time.^21^, ^22^

For that reason, topical use can be effective through contact with clot surface,^8^ but in a previous study, the favorable effect of tranexamic acid on bleeding and surgical field in different periods of time tended to diminish with the passage of time due to the reduction in local concentration.^7^ Thus, operative time would be influenced by these time-dependent changes more significantly than overall operative bleeding and quality of surgical field. As an additional explanation, previous meta-analysis also demonstrated that operative time did not significantly differ between the tranexamic acid group and control. In previous study,^15^ authors explained that the small sample size in this outcome, only two RCTs for systemic administration, would have little statistical significance. We also enrolled three RCTs for operative time, which could explain the discrepancy with result and expectation.

The most common side effects of tranexamic acid are gastrointestinal adverse effects, involving postoperative nausea and vomiting, and systemic administration can lead to severe hypotension.^18^ In addition, the risk of thromboembolism has traditionally raised concerns about its use.^19^, ^20^ In this study, the topical administration of tranexamic acid did not cause a significant change in intra-operative blood pressure or the incidence of postoperative emetic effect compared to the control group. Many studies have suggested that the adverse effects of tranexamic acid are dose-dependent in intravenous administration and rare in local application.^8^, ^9^, ^10^, ^23^ Our meta-analysis also showed that this agent did not induce the thromboembolism accident at all, a tendency similar to that of the control. The coagulation profile of the treatment group was similar to that of the control group, which may be consistent with previous results and support the incidence of this study.^4^, ^7^ The main reason for the lower risk of adverse effects would be the direct and local contact to the working field without systemic absorption.^8^ Although topical application would be absorbed systemically to some degree, and in our study plasma tranexamic acid concentrations were not measured and indirect measurements of the coagulation profile (PT and PTT) were unchanged. Previously, topical application as a mouthwash was reported not to significantly affect plasma level.^24^ These results could support that topical application in sinus surgery is a safe and effective management method for operative bleeding.

Our study had some limitations. First, we tried to maximize the enrolled studies by reviewing multiple databases, but we only enrolled a few studies. Therefore, additional research is needed to better estimate treatment effects and outcomes. Second, wide ranges of dosages and diverse application methods, such as irrigation and packing, were used because there is yet to be a consensus on actual methodology. Detailed regimens, including other systemic agents could also have differed among studies. In this study, the enrolled studies regarding topical effect applied the use of topical agents as perioperative preparation in both study and control groups. In order to increase the number of available comparisons, we included studies that evaluate the effects of tranexamic acid in patients who underwent different administration protocols. This fact may have contributed to some of the heterogeneity observed in this study. However, we tried to rule out factors other than tranexamic acid from our consideration with statistical methods (standardized mean difference) because only difference between study and control groups in each study was whether or not tranexamic acid was administrated topically. In our meta-analysis, we statistically calculated this additional effect derived from topical tranexamic acid in each study and quantitatively combined the results. The measurement could minimize the influence of other factors of endoscopic sinus surgery and mainly assess the effect of intervention. Theoretical quantification could be an inherent drawback of a meta-analysis but to our best knowledge, this is the first study to try to gain insight into local hemostatic effect of tranexamic acid itself. Considering these limitations, a large-sample, randomized, controlled clinical study should be performed to provide further evidence on the efficacy of the topical administration of tranexamic acid in endoscopic sinus surgery.

Furthermore, most of the studies used normal saline for control group but in real surgery setting, other types of topical or systemic hemostatic agents are frequently used. Thus, in future research and analysis, we think control group could be designed in a way to compare tranexamic acid application and other known methods to control intraoperative bleeding. Nevertheless, in consideration of the low morbidity and convenience of these treatments, it is reasonable to conclude that topical usage of tranexamic acid is applicable for the majority of patients that undergo nasal surgery.

## Conclusion

The authors concluded that the topical tranexamic acid application may have several positive effects on intraoperative bleeding and operative field in patients undergoing nasal surgery. Particularly, there were side effects similar to postoperative vomiting compared to the control group. There was no incidence of severe complication such as thromboembolism in the included studies. However, given the number of studies enrolled and the various methods of administration, large scale RCTs employing standardized dosing methods are required.

## Funding

This research was supported by Basic Science Research Program through the National Research Foundation of Korea (NRF) funded by the Ministry of Education (2018R1D1A1B07045421).

## Conflicts of interest

The authors declare no conflicts of interest.
